# Development of a Yoga Program for Type-2 Diabetes Prevention (YOGA-DP) Among High-Risk People in India

**DOI:** 10.3389/fpubh.2020.548674

**Published:** 2020-11-17

**Authors:** Kaushik Chattopadhyay, Pallavi Mishra, Nandi Krishnamurthy Manjunath, Tess Harris, Mark Hamer, Sheila Margaret Greenfield, Haiquan Wang, Kavita Singh, Sarah Anne Lewis, Nikhil Tandon, Sanjay Kinra, Dorairaj Prabhakaran

**Affiliations:** ^1^Division of Epidemiology and Public Health, University of Nottingham, Nottingham, United Kingdom; ^2^The Nottingham Centre for Evidence-Based Healthcare: A Joanna Briggs Institute Centre of Excellence, Nottingham, United Kingdom; ^3^Centre for Chronic Disease Control, New Delhi, India; ^4^Swami Vivekananda Yoga Anusandhana Samsthana, Bengaluru, India; ^5^Population Health Research Institute, St. George's University of London, London, United Kingdom; ^6^Division of Surgery and Interventional Science, Institute Sport Exercise and Health, University College London, London, United Kingdom; ^7^Institute of Applied Health Research, University of Birmingham, Birmingham, United Kingdom; ^8^Department of Endocrinology, Metabolism and Diabetes, All India Institute of Medical Sciences, New Delhi, India; ^9^London School of Hygiene and Tropical Medicine, London, United Kingdom

**Keywords:** Yoga, prevention, prediabetes, lifestyle, physical activity, diet

## Abstract

**Introduction:** Many Indians are at high-risk of type-2 diabetes mellitus (T2DM). Yoga is an ancient Indian mind-body discipline, that has been associated with improved glucose levels and can help to prevent T2DM. The study aimed to systematically develop a Yoga program for T2DM prevention (YOGA-DP) among high-risk people in India using a complex intervention development approach.

**Materials and Methods:** As part of the intervention, we developed a booklet and a high-definition video for participants and a manual for YOGA-DP instructors. A systematic iterative process was followed to develop the intervention and included five steps: (i) a systematic review of the literature to generate a list of Yogic practices that improves blood glucose levels among adults at high-risk of or with T2DM, (ii) validation of identified Yogic practices by Yoga experts, (iii) development of the intervention, (iv) consultation with Yoga, exercise, physical activity, diet, behavior change, and/or diabetes experts about the intervention, and (v) pretest the intervention among Yoga practitioners and lay people (those at risk of T2DM and had not practiced Yoga before) in India.

**Results:** YOGA-DP is a structured lifestyle education and exercise program, provided over a period of 24 weeks. The exercise part is based on Yoga and includes Shithilikarana Vyayama (loosening exercises), Surya Namaskar (sun salutation exercises), Asana (Yogic poses), Pranayama (breathing practices), and Dhyana (meditation) and relaxation practices. Once participants complete the program, they are strongly encouraged to maintain a healthy lifestyle in the long-term.

**Conclusions:** We systematically developed a novel Yoga program for T2DM prevention (YOGA-DP) among high-risk people in India. A multi-center feasibility randomized controlled trial is in progress in India.

## Introduction

India has the second-largest type-2 diabetes mellitus (T2DM) population in the world, a disorder with major health and socioeconomic consequences (1). More than 77 million Indians are at high-risk of T2DM—their blood glucose levels are higher than normal but lower than the established threshold for T2DM itself (2). These people are more likely to develop T2DM and its complications than those with normal blood glucose levels (3). Unhealthy lifestyle (i.e., physical inactivity and unhealthy diet) is a major risk factor for T2DM (3). Physical activity levels are low among Indians (4). Similarly, unhealthy diets, high in fat (especially saturated fat) and low in fiber, are more prevalent among Indians (5, 6). Screening of people who are at high-risk of T2DM, followed by an effective lifestyle intervention is a cost-effective approach that can normalize blood glucose levels and has other health benefits (3, 7, 8).

Health interventions should be informed by and compatible with the sociocultural expectations of people and their health beliefs (9). The prevention and management of chronic diseases like T2DM using traditional Indian therapies have been prioritized by the Indian government (10). Yoga, an ancient Indian mind-body discipline, covers not only physical activity but also a healthy diet (11). Many different styles of Yoga are undertaken, focusing on similar core elements of physical, mental, and spiritual practices. No particular style of Yoga is necessarily better or more authentic than the others (12). Indians usually have high acceptability of Yoga because it fits their health beliefs and culture (13, 14). Generally, it uses a gentle approach, is easy to learn, is safe, requires a low to moderate level of guidance, is inexpensive to maintain, and can be practiced indoors and outdoors (13). It can be practiced by older adults and those with a wide range of comorbidities (12, 13). As a form of physical activity, some of the Yogic practices are of low-intensity (<3.5 kcal/min) and some are of moderate-intensity (3.5–7.0 kcal/min) (12, 15). For example, Surya Namaskar (sun salutation exercises) burns about 3.8–6.7 kcal/min (16, 17). Yoga is also a muscle-strengthening activity (12). Thus, it can contribute to the aim of routine lifestyle advice given to people at high-risk of T2DM to prevent it.

The beneficial effects of Yoga on T2DM-related risk profiles appear to occur via two main pathways. First, by reducing the activation and reactivity of the sympathoadrenal system and the hypothalamic-pituitary-adrenal axis and by promoting the feelings of well-being, Yoga may alleviate the effects of stress and foster multiple positive downstream effects on the neuroendocrine status, metabolic function, and related systemic inflammatory responses (18). Second, by directly stimulating the vagus nerve, Yoga may enhance the parasympathetic activity and lead to positive changes in the cardiovagal function, mood, energy state, and related neuroendocrine, metabolic, and inflammatory responses (18). In addition, Yoga may lead to weight loss, which itself lowers the risk of T2DM (18).

The beneficial effects of Yoga on T2DM-related outcomes in T2DM (as adjuvant therapy) and metabolic syndrome have been reported in several systematic reviews of clinical trials (19–22). For example, a review of 44 randomized controlled trials (RCTs), conducted among T2DM, metabolic syndrome, or healthy participants (*n* = 3,168), found that Yoga improves blood glucose levels compared to usual care or no intervention (mean difference = −0.45%; 95% confidence interval = −0.87 to −0.02), without any major safety issues (19). However, most of the included studies were short-term (≤ 3 months) and were often associated with considerable methodological limitations, such as small sample sizes in treatment groups, resulting in lack of statistical power for outcome assessment, and lack of blinding of outcome assessors, leading to potential analysis bias. In addition, some of the relevant previous studies have not described the intervention in detail, and it is difficult to replicate successful interventions. Most studies have not reported the intervention development process. It is hard to know whether these interventions were carefully thought out (e.g., their safety and acceptability) and comprehensive in their development. Thus, it is difficult to select (and replicate) one successful intervention over the other. Another selection barrier is their heterogeneous contents (i.e., different Yogic practices were included in these interventions), which needed to be summarized for utilization in T2DM prevention. Thus, our study aimed to address these issues by systematically developing a Yoga program for T2DM prevention (YOGA-DP) among high-risk people in India.

## Materials and Methods

We followed a systematic iterative process to develop the intervention, guided by the UK's Medical Research Council (MRC) guidance on developing and evaluating complex interventions and Sherman's guideline for developing Yoga interventions for RCTs (9, 23). The template for intervention description and replication (TIDieR) checklist and guide were used to report the intervention (24). As part of the intervention, we developed a booklet and a high-definition video for participants and a manual for YOGA-DP instructors. An external filmmaking company was hired to convert the booklet (Yoga part) into a video to aid audio-visual learning. It was decided to use USB flash drives (having a compressed video—2.08 gigabyte (GB) in MPEG-4 Part 14 (MP4) file format) after discussing the technological advancements as well as accessibility issues in India with the relevant stakeholders (e.g., those in the field of film making). All these are available in English and two other Indian languages, Hindi and Kannada.

**Figure 1:**
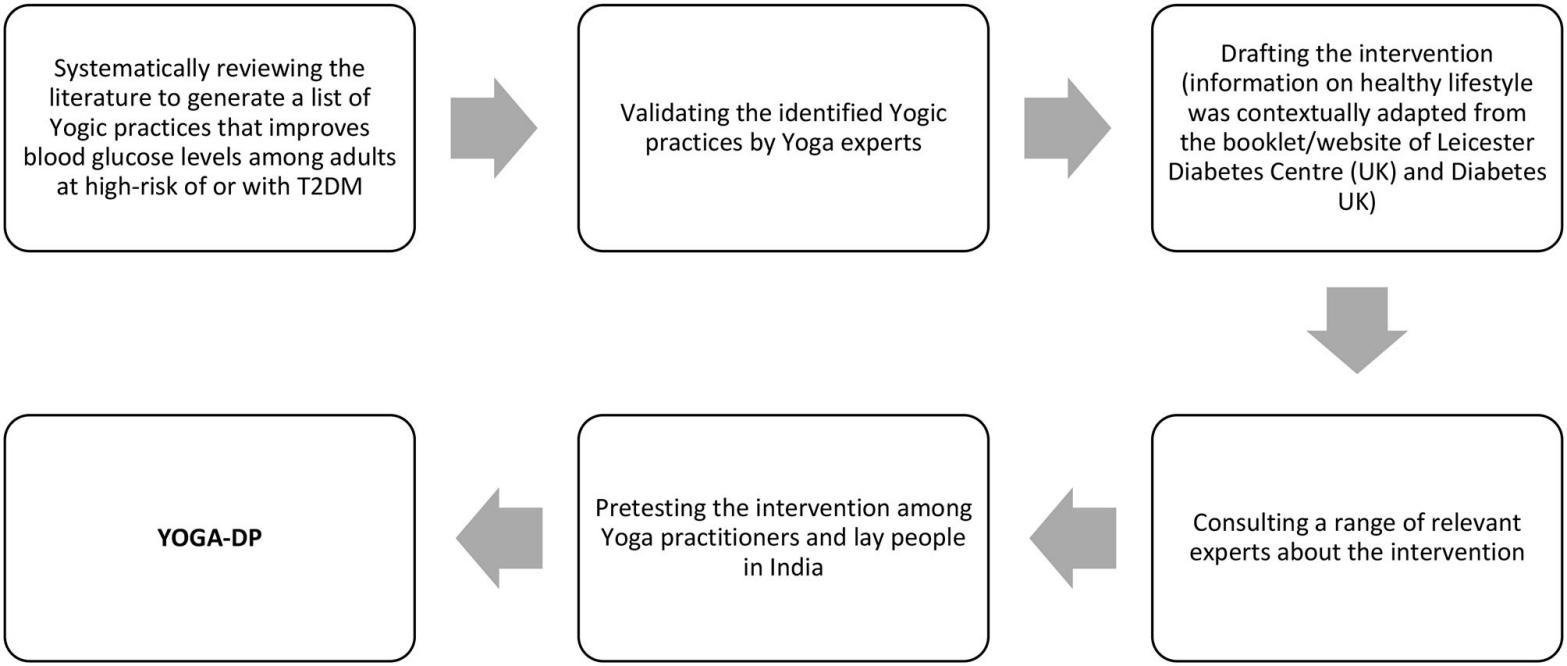
Development process of YOGA-DP.

Figure 1 shows the development process of YOGA-DP, which consisted of five steps:

(1) We conducted a systematic review of scientific literature to generate a list of Yogic practices that improves blood glucose levels among adults at high-risk of or with T2DM. The PROSPERO protocol informed the systematic review process (PROSPERO registration number CRD42018097216) (25). The process was guided by the Preferred Reporting Items for Systematic Reviews and Meta-Analyses (PRISMA) documents (26). Two Joanna Briggs Institute (JBI) accredited systematic reviewers (KC/HW) were involved in the process and independently screened the titles and abstracts and full-text of studies, assessed the methodological quality of studies, and extracted data from the studies. Any disagreements between them were resolved through discussion.

### Inclusion and Exclusion Criteria

Population, intervention, comparator, and outcome: Studies targeting adults (≥18 years) at high-risk of or with T2DM were eligible. The diagnosis was based on blood glucose levels. Studies comparing Yoga intervention to no or any intervention and reporting successful Yoga interventions were eligible i.e., Yogic practices which improved blood glucose levels. At least one of the following needed to be statistically significant in the Yoga intervention group as compared to the control group: fasting blood glucose (FBG), postprandial blood glucose (PPBG), or glycated hemoglobin (HbA1c). Studies reporting at least one Yogic practice (i.e., Asana (Yogic pose), Pranayama (breathing practice), or Dhyana (meditation) and relaxation practice), based on classical Yoga texts, were eligible. Studies were excluded if they did not report the Sanskrit name of the Yogic practice. No restrictions were made regarding the frequency or duration of the Yoga intervention. Studies on multimodal interventions that included Yoga amongst others were excluded. Studies allowing individual co-interventions were eligible i.e., studies allowing participants to continue their individual treatment were not excluded as long as all the study groups were allowed to do so.

Study design: Only RCTs were eligible.

Language of publication: No language restrictions were applied.

### Search Strategy

(a) Using existing full-text peer-reviewed systematic reviews as a starting point for identifying the relevant RCTs (and interventions), systematic reviews were searched on PubMed from its inception date to 7th May 2018. The systematic review authors (reviewers) searched a range of databases, the reference list of identified original and review articles, and the table of contents of relevant journals, trial registers, proceedings, and abstracts from relevant symposiums, conferences, and colloquiums. These systematic review authors also approached the relevant experts. The search strategy used was: (Yoga[MeSH] OR Yoga^*^[tiab] OR Yogi^*^[tiab] OR Asana^*^ [tiab] OR Pranayam^*^ [tiab] OR Dhyan^*^ [tiab]) AND systematic[sb].

(b) To supplement the above step, we searched on PubMed to directly identify any full-text peer-reviewed RCTs that were published between 1st January 2015 and 7th May 2018. The search strategy used was: (Yoga[MeSH] OR Yoga^*^[tiab] OR Yogi^*^[tiab] OR Asana^*^ [tiab] OR Pranayam^*^ [tiab] OR Dhyan^*^ [tiab]) AND random^*^[mp] NOT systematic[sb].

(c) We also searched IndMED and Google Scholar for additional systematic reviews and RCTs, using different words for Yoga, prediabetes, diabetes, systematic review, and RCT. For Google Scholar, the advanced search tool was used (with all of the words in the title of the article) and excluded patents and citations. The reference list of all the relevant RCTs was also screened for additional RCTs.

### Screening and Full-Text Reading

Following the search, all identified citations were collated and uploaded into EndNote X8.2, a reference management software (27). Titles and abstracts were screened for eligibility using the inclusion and exclusion criteria. The full-texts of studies identified as potentially eligible or those without an abstract were retrieved and assessed against the inclusion and exclusion criteria. The full-texts of studies that did not meet the inclusion criteria were excluded, and the reasons for exclusion were reported (Supplementary Table 1).

### Methodological Quality Assessment

In terms of methodological quality of the included RCTs, the following were assessed: randomization method, allocation concealment, blinding of outcome assessors, and withdrawals and dropouts. The Jadad score was also calculated. The Jadad score ranges from zero to five points—two points are given for randomization, two points for blinding, and one point for dropouts (28). A low-quality study receives a score of two points or less, and a high-quality study receives a score of at least three points (28). The Jadad score is easy to use, contains many of the important elements that have empirically been shown to correlate with bias, and has known reliability and external validity (29). Data were extracted and synthesized from all the included RCTs, regardless of their methodological quality.

### Data Extraction

Study characteristics of the included RCTs and the intervention details were extracted using a standardized data extraction tool.

### Data Synthesis

Narrative data synthesis was conducted as the aim of the systematic review was to generate a list of Yogic practices that improves blood glucose levels among adults at high-risk of or with T2DM.

(2) We required at least 40 Yoga experts (41 responders) to validate each of the identified Yogic practices, based on Lawshe's content validity ratio (CVR) formula (30). Highly qualified and experienced Yoga practitioners and/or Yoga researchers with an interest in diabetes (including those with high-level authority) were purposively selected in India for this purpose. One of the study authors (NKM) is a key person in the field of Yoga, and this helped us to gain access to these Yoga experts. A questionnaire with all the identified practices was administered electronically through email, and they marked the content validity of each practice on a three-point scale (zero = not necessary, one = useful but not essential, two = essential), taking into account the safety aspect. CVR was calculated for each practice but only those with CVR ≥ 0.29 were included in the intervention (30, 31).

$$\begin{array}{r} \text{CVR}=\text{(ne-N/2)}/\text{(N/2)} \end{array}$$

Where,

ne = number of experts indicating “essential”

N = total number of experts.

(3) We drafted the intervention, with texts and pictorials. With permission, the information on being at high-risk of T2DM and how to prevent T2DM by being more physically active, keeping a healthy weight, eating less fat (especially saturated fat), and eating more fiber was extracted from an existing booklet of Leicester Diabetes Centre (UK) and the Diabetes UK website and adapted to the Indian context (32, 33). Any traditional advice which is based on anecdotal (or contradictory) evidence was not included in the intervention e.g., dietary advice to consume ghee, a type of clarified butter composed almost entirely of fat, especially saturated fat.

(4) We conducted a consultation on the intervention with 16 experts in Yoga, exercise, physical activity, diet, behavior change, and/or diabetes from India and the UK. Like step number two, we used our multidisciplinary team's contact to approach these experts. The intervention was shared with them through email, and they further reviewed its structure and content, especially from the safety point of view. Their feedback (through email) was used to improve it.

(5) We pre-tested the intervention among eight Yoga practitioners (teachers) and six lay people (those at risk of T2DM and had not practiced Yoga before) at Swami Vivekananda Yoga Anusandhana Samsthana (S-VYASA), India. The objectives were to identify any difficulties they had in reading and understanding the intervention (i.e., comprehension of content/instructions) and to explore the acceptability of the intervention and ways to promote its uptake and adherence. In addition, Yoga practitioners were asked about the overall sequence and flow of the intervention and any difficulties they had in delivering the intervention, especially within the intended timeframe. They were purposively selected to ensure representation of diversity by age and sex. The intervention was shared with them prior to the following activities—to enable them to read and understand it in their free time:

(a) Yoga practitioners: A face-to-face meeting was held with them.

(b) Lay people: A Yoga session, based on the intervention, was delivered to them by two Yoga practitioners (one male and one female). This was followed by a face-to-face meeting with them.

With consent, the feedback was noted and digitally-recorded. Their feedback was used to finalize the intervention.

## Results

The results describe the outcome of each step of the intervention development process.

(1) A total of nine RCTs were included in our systematic review. Supplementary Figure 1 shows the flowchart depicting the search and screening process of systematic reviews and RCTs.

(a) 501 systematic review records were identified until 7th May 2018. 468 records were excluded at the title and abstract screening stage. The full-texts of 33 systematic reviews were assessed for eligibility and 21 were excluded at this stage. The remaining 12 systematic reviews were included containing potential RCTs (19, 21, 22, 34–42). The full-texts of 67 potential RCTs were assessed for eligibility and 58 were excluded at this stage. The remaining eight RCTs (two similar articles from the same RCT were published in two different journals) were included in our systematic review (43–51).

(b) To supplement the above step, 384 RCT records were directly identified between 1st January 2015 and 7th May 2018. 374 records were excluded at the title and abstract screening stage. The full-texts of 10 RCTs were assessed for eligibility and nine were excluded at this stage. The remaining one RCT was included in our systematic review (52).

Supplementary Tables 2, 3 report the study characteristics and critical appraisal of the nine included RCTs, respectively. Briefly, all the RCTs were conducted in India. The sample size ranged from 30 to 337. Yoga was an adjuvant therapy in all the RCTs—all the RCTs were conducted among T2DM patients and one study also included people with prediabetes. The improvement in blood glucose levels was measured using FBG, PPBG, and/or HbA1c tests. One RCT mentioned that no adverse event occurred and four reported limited information on adverse events. The Jadad score of only four RCTs was high. Only four RCTs mentioned the allocation concealment (two provided only limited information). Table 1 reports the intervention details of the included RCTs. More specifically, 58 Yogic practices that improve blood glucose levels among adults at high-risk of or with T2DM were identified.

**Table 1:** Intervention details of the included RCTs.

**References**	**Intervention development information**	**Yoga sessions: frequency and duration**	**Yoga sessions: delivery (place and person)**	**Shithilikarana Vyayama**	**Surya Namaskar**	**Asana**	**Pranayama**	**Dhyana and relaxation practice**	**Extra features**
Agrawal et al. (43)	NS	60 min/day X 5–7 days/week X 12 weeks	At the hospital, NS	Yes	Yes	Paschimottanasana, Ardhamatsyendrasana, Uttanapadasana, Sarvangasana, Matsyasana	Yes (NS)	Kayotsarga, Preksha meditation including Anupreksha	
Nagarathna et al. (44)	Developed by experts including Yoga, based on the knowledge culled out from Yoga scriptures (Patanjali Yoga Sutras, Bhagavad Gita, and Mandukya Karika)	60 min/day X 5 days/week X 12 weeks (and then till 9 months: 1 session (of 120 min)/week and 60 min/day X 7 days/week self-practice at home)	NS, by Yoga instructor	Yes	Yes	Parivrttatrikonasana, Vakrasana, Ardhamatsyendrasana, Ustrasana, Hamsasana, Mayurasana, Bhujangasana, Dhanurasana, Sarvangasana, Matsyasana, Padahastasana, Ardhachakrasana, Trikonasana, Pavanamuktasana, Shavasana	Vibhagiya, Ujjayi, Nadishodhana, Sheetali, Shitkari, Bhramari, Kapalbhati	Nadanusandhana (A Kara, U Kara, M Kara, and AUM chanting)	Self-reporting of Yoga practice at home (types and min/day), pre-recorded Yoga instruction audiotape for participants
Vaishali et al. (45, 46)	NS	45–60 min/day X 6 days/week X 12 weeks	At the hospital, by Yoga instructor			Vajrasana, Suptavajrasana, Tadasana, Padahastasana, Trikonasana, Paravakonasana, Trikonasana, Vakrasana, Pavanamuktasana, Bhujangasana, Shalabhasana, Shavasana	Ujjayi, Anulom Vilom		Weekly talks by a motivated participant on perceived benefits and personal experiences of regular Yoga practice, followed by group discussions among participants for enhancing adherence
Yadav (47)	NS	? X 12 weeks	NS, NS			Poornabhujangasana, Dhanurasana, Baddhapadmasana, Kukkutasana, Halasana			
Kumar and Kalidasan (48)	NS	50 min/day X 6 days/week X 12 weeks (morning sessions)	At a Yoga center?, by Yoga instructor	Yes		Tadasana, Konasana, Padahastasana, Piraiasana, Yoga Mudrasana (Padmasana, Vajrasana, Sukhasana), Janusirsasana, Vakrasana, Ustrasana, Makarasana, Pavanamuktasana, Uttanapadasana, Naukasana, Bhujangasana, Ardhashalabhasana, Poornashalabhasana, Dhanurasana, Shavasana			
Kumpatla et al. (49)	Based on earlier reports	30 min/day X 7 days/week X 12 weeks (one training session in the morning and then self-practice at home)	At the hospital, by Yoga instructor			Vakrasana, Paschimottanasana, Mandukasana, Uttanapadasana, Naukasana, Bhujangasana, Trikonasana			Regular phone calls for encouraging self-practice at home, Yoga booklet for participants
Sharma et al. (50)	NS	45–60 min/day X 5 days/week X 12 weeks (morning sessions on empty stomach)	At the hospital, by Yoga instructor			Trikonasana, Tadasana, Sukhasana, Padmasana, Mandukasana, Paschimottanasana, Ardhamatsyendrasana, Pavanamuktasana, Bhujangasana, Vajrasana, Dhanurasana, Shavasana	Yes (NS)		
Singh et al. (51)	NS	? min/day X 7 days/week X 2 weeks (and then till 6 months: once/month supervision at the delivery center? and self-practice at home)	NS, by Yoga instructor	Yes	Yes	Tadasana, Trikonasana, Vajrasana, Padmasana, Ardhamatsyendrasana, Paschimottanasana, Bhujangasana, Dhanurasana, Halasana, Naukasana, Shavasana	Bhastrika, Kapalbhati, Anulom Vilom, Bhramari		Self-reporting of Yoga practice at home (types/day and lapse), requesting family/carer to accompany participant during sessions and to countersign once participant finishes self-practice at home, weekly phone call to participant and family/carer for monitoring adherence and knowing difficulties, Yoga booklet for participants
Keerthi et al. (52)	Formulated in accordance with guidelines of Morarji Desai National Institute of Yoga, India	45 min X 3 days/week X 12 weeks (and self-practice at home)	At the hospital, by Yoga instructor	Yes	Yes	Padahastasana, Konasana, Vakrasana, Ardhamatsyendrasana, Paschimottanasana, Shalabhasana, Dhanurasana, Pavanamuktasana, Ardhahalasana, Saralmatsyasana, Tadasana, Katichakrasana, Shavasana	Nadishodhana, Bhramari, Chandranadi	Nadanusandhana (A Kara, U Kara, M Kara, and AUM chanting), Yoga Nidra	Attendance documentation of Yoga sessions, regular phone calls for monitoring self-practice at home, Yoga booklet for participants

*NS, Not specified; ?, Unclear*.

(2) Table 2 reports the validation of the identified Yogic practices. Out of the 58 identified Yogic practices, 31 were included in the intervention, namely, Shithilikarana Vyayama (loosening exercises), Surya Namaskar, 22 Asana (six standing poses, seven sitting poses, four lying poses-front/prone, and five lying poses-back/supine), five Pranayama, and two Dhyana and relaxation practices. There was one exception—Ardhaustrasana, a simplified form of Ustrasana (a sitting pose included in the intervention), was additionally included in the intervention as recommended by the Yoga experts during the validation work.

**Table 2:** Validation of the identified Yogic practices.

**Yogic practices**	**CVR**	**Additional reason for exclusion**	**Included in the intervention?**
Shithilikarana Vyayama	0.71		Yes
Surya Namaskar	0.80		Yes
Ardhachakrasana	0.61		Yes
Katichakrasana	0.90		Yes
Padahastasana	0.51	Part of Surya Namaskar	No
Piraiasana	−0.56		No
Tadasana	0.37		Yes
Konasana	0.90		Yes
Paravakonasana	0.37		Yes
Trikonasana	0.56		Yes
Parivrttatrikonasana	0.27		No
Ardhamatsyendrasana	0.95		Yes
Janusirsasana	0.51		Yes
Kukkutasana	−0.95		No
Mandukasana	0.90		Yes
Padmasana	−0.02		No
Baddhapadmasana	−0.37		No
Paschimottanasana	0.66		Yes
Sukhasana	0.22		No
Ustrasana	0.66		Yes
Vajrasana	0.56		Yes
Vakrasana	1.00		Yes
Yoga Mudrasana 1 (Padmasana)	0.07		No
Yoga Mudrasana 2 (Vajrasana)	0.27		No
Yoga Mudrasana 3 (Sukhasana)	0.12		No
Bhujangasana	0.95	Part of Surya Namaskar	No
Poornabhujangasana	−0.71		No
Dhanurasana	0.80		Yes
Hamsasana	−0.51		No
Makarasana	0.66		Yes
Mayurasana	−0.51		No
Shalabhasana/Poornashalabhasana	0.32		Yes
Ardhashalabhasana	0.51		Yes
Halasana	0.12		No
Ardhahalasana	0.61		Yes
Matsyasana	0.17		No
Saralmatsyasana	0.37		Yes
Naukasana	0.66		Yes
Pavanamuktasana	0.95		Yes
Suptavajrasana	−0.22		No
Sarvangasana	0.07		No
Uttanapadasana	0.61		Yes
Shavasana	0.90	Similar to Yoga Nidra	No
Anulom Vilom Pranayama	0.80	Similar to Nadishodhana Pranayama	No
Nadishodhana Pranayama	0.85		Yes
Chandranadi Pranayama	−0.02		No
Bhastrika Pranayama	0.37		Yes
Kapalbhati Pranayama	0.56		Yes
Bhramari Pranayama	0.80		Yes
Sheetali Pranayama	−0.17		No
Shitkari Pranayama	−0.27		No
Ujjayi Pranayama	−0.17		No
Vibhagiya Pranayama	0.51		Yes
Nadanusandhana (A Kara, U Kara, M Kara, and AUM chanting)	0.76	AUM chanting removed on religious grounds	Partially
AUM chanting	0.46	On religious grounds	No
Kayotsarga	0.80	Part of Yoga Nidra	No
Preksha meditation including Anupreksha	0.32	Part of Yoga Nidra	No
Yoga Nidra	0.76		Yes

(3–5) The intervention is for adults (18–74 years) who are at high-risk of T2DM and are currently safe to do physical activity, determined by the Physical Activity Readiness Questionnaire (PAR-Q)/clinician. The intervention is not suitable for pregnant women; people with chest pain, a heart condition, or any serious or uncontrolled medical condition; or people who have recently undergone surgery. People with high blood pressure are required to check first with their clinician that their blood pressure is well-controlled before taking part in the intervention.

Tables 3, 4 report the structure of YOGA-DP and structure and content of the Yoga sessions, respectively. The intervention is a structured lifestyle education and exercise program, provided over a period of 24 weeks. The exercise part is based on Yoga and includes 32 Yogic practices, namely, Shithilikarana Vyayama, Surya Namaskar, 23 Asana (six standing poses, eight sitting poses, four lying poses-front/prone, and five lying poses-back/supine), five Pranayama, and two Dhyana and relaxation practices. Once participants complete the intervention, they are strongly encouraged to maintain a healthy lifestyle in the long-term, using the intervention booklet and video. It should be noted that, initially, we planned 52 supervised center-based group sessions (75 min/session X two sessions/week X 26 weeks). However, the intervention structure was modified after the consultation work to enhance intervention uptake and adherence i.e., based on the recommendations of experts in step number four. In the first instance, 75 min/session might appear a huge amount of time for the participants to commit to, and thus, we designed 45 min session in weeks 1–4 to avoid any physical or mental exhaustion and to gradually build their fitness. Second, to avoid the excessive burden of attending center-based sessions, weeks 13–24 comprise of one center-based session every 4 weeks and self-practice at home (using the intervention booklet and video) is recommended from week 13 onwards. Initial supervised center-based sessions are deemed to be appropriate for wider use in India due to the low levels of literacy. Similarly, initial group sessions are deemed to be appropriate for benefits from shared experiences and peer support. To improve intervention uptake and adherence, the group Yoga sessions are run locally (e.g., at Yoga centers and community centers) and at different time points of the day (with evening and weekend sessions), and participants can join as per their convenience. Some of their local travel costs for attending these sessions are reimbursed. A family member or someone close to the participant is invited to join them in these sessions. The intervention is delivered by YOGA-DP instructors—qualified and experienced Yoga teachers with formal training provided on the program, and they can speak the local languages. Female instructors are available for female participants.

**Table 3:** Structure of YOGA-DP.

**Week**	**Group Yoga sessions delivered by YOGA-DP instructors**	**Self-practice of Yoga at home using YOGA-DP booklet and a video**	**Extra features**
1–4 (month 1)	At least two sessions of 45 min per week. An attendance register is kept.	–	At the first session, the instructor is giving participants part one of our program booklet. This gives them information about being at high-risk of T2DM and how to prevent T2DM (i.e., by being more physically active, keeping a healthy weight, eating less fat (especially saturated fat), and eating more fiber).
5–12 (month 2–3)	At least two sessions of 75 min per week. An attendance register is kept.	–	At the last session, the instructor is giving participants part two of our program booklet and a video. These give them information on Yoga practice to prevent T2DM. Also, a Yoga diary and a non-slippery Yoga mat are provided for self-practice of Yoga at home.
13–24 (month 4–6)	At least one session of 75 min every 4 weeks. An attendance register is kept.	At least two sessions of 75 min per week. Participants are given the Yoga diary to record their Yoga practice (types and minutes).	The instructor is phoning participants every week to offer support and help and to troubleshoot any problems.
25+ (month 7+)	–	At least two sessions of 75 min per week. Participants are given the Yoga diary to record their Yoga practice (types and minutes).	–

**Table 4:** Structure and content of the Yoga sessions.

**Yogic practices**	**Week 1– Each session should last for 45 min with the time split as follows:**	**Week 5+ Each session should last for 75 min with the time split as follows:**	**Details**
Shithilikarana Vyayama	Around 5 min	Around 5 min	(1) Neck rotation *30 s* (2) Shoulder rotation *30 s* (3) Elbow flexion and extension *30 s* (4) Wrist rotation *30 s* (5) Finger movement *30 s* (6) Waist rotation *30 s* (7) Knee flexion and extension *1 min* (8) Ankle rotation *1 min* (9) Toe movement *30 s*
Surya Namaskar	–	Around 15 min	The below mentioned 12 steps constitute one set of Surya Namaskar. To complete one round of Surya Namaskar, participants need to repeat these 12 steps on the other side of their body (i.e., by extending their left leg behind in step number 4 and bringing their left leg forward in step number 9). Initially, they should practice Surya Namaskar at a slower pace. Only with practice over some time, they may try to do 12 rounds of it at a faster pace for around 15 min (i.e., a couple of seconds per step). (1) Pranamasana (prayer pose) (2) Hastauttanasana (raised arms pose) (3) Padahastasana (hands to feet pose) (4) Ashwa Sanchalanasana (equestrian pose) (5) Dandasana (stick pose) (6) Ashtanga Namaskara Asana (salute with eight parts) (7) Bhujangasana (cobra pose) (8) Parvatasana (mountain pose) (9) Ashwa Sanchalanasana (equestrian pose) (10) Padahastasana (hands to feet pose) (11) Hastauttanasana (raised arms pose) (12) Pranamasana (prayer pose)
Asana	Around 15 min	Around 30 min	Two-sided poses (right and left) are to be practiced for about 3 min (1.5 min on each side) and central-positioned poses are to be practiced for about 1.5 min. In each session, the Yogic poses are selected from the list below to prevent boredom from the similarity of routine. Advanced Yogic poses are introduced from week 5 onwards, for example, Konasana (angle pose), Trikonasana (triangle pose), Paravakonasana (lateral angle pose), Ardhaustrasana (half camel pose), Ustrasana (camel pose), Dhanurasana (bow pose), and Naukasana (boat pose). (A) Standing poses (1) Tadasana (palm tree pose) *1.5 min* (2) Ardhachakrasana (half wheel pose) *1.5 min* (3) Katichakrasana (waist wheel pose) *3 min* (4) Konasana (angle pose) or Trikonasana (triangle pose) or Paravakonasana (lateral angle pose): alternatively *3 min* (B) Sitting poses (1) Vajrasana (adamant pose) *1.5 min* (2) Mandukasana (frog pose) *1.5 min* (3) Ardhaustrasana (half camel pose) or Ustrasana (camel pose): alternatively *1.5 min* (4) Vakrasana (twisted pose) or Ardhamatsyendrasana (half spinal twist pose): alternatively *3 min* (5) Paschimottanasana (seated forward bend pose) or Janusirsasana (head to knee pose): alternatively *1.5 or 3 min, respectively* (C) Lying poses- front/prone (1) Ardhashalabhasana (half locust pose) or Poornashalabhasana (full locust pose): alternatively *3 or 1.5 min, respectively* (2) Dhanurasana (bow pose) *1.5 min* (3) Makarasana (crocodile pose) *1.5 min* (D) Lying poses- back/supine (1) Uttanapadasana (raised legs pose) or Ardhahalasana (half plow pose): alternatively *1.5 min* (2) Pavanamuktasana (wind relieving pose) *1.5 min* (3) Naukasana (boat pose) *1.5 min* (4) Saralmatsyasana (easy fish pose) *1.5 min*
Pranayama	Around 13 min	Around 13 min	(1) Vibhagiya Pranayama (sectional breathing) *4 min* (2) Nadishodhana Pranayama (alternate nostril breathing) *3 min* (3) Kapalbhati Pranayama (skull shining breathing) or Bhastrika Pranayama (bellow breathing): alternatively *3 min* (4) Bhramari Pranayama (bee breathing) *3 min*
Dhyana and relaxation practices	Around 12 min	Around 12 min	In each session, the following Dhyana and relaxation practices are to be done in a darkened room. (1) A Kara chanting, U Kara chanting, and M Kara chanting *3 min* (2) Yoga Nidra (Yogic sleep) *9 min*

## Discussion

We report the systematic development of a novel Yoga program for T2DM prevention (YOGA-DP) among high-risk people in India. The duration of our intervention (24 weeks) is longer than many other Yoga interventions (43, 45–50, 52). Even after formally completing the intervention, participants are strongly encouraged to maintain a healthy lifestyle in the long-term, using the intervention booklet and video. The long-term maintenance of a healthy lifestyle is required, not just for preventing T2DM but also for overall health (3). However, lifestyle interventions' poor uptake and adherence (especially over the long-term) are well-recognized and can negatively affect the effectiveness of these interventions (53). This is the reason we incorporated multiple strategies to enhance uptake and adherence to our intervention. Some of these have already been used in previous successful studies (44–46, 49, 51, 52) and some came up during the intervention development process (e.g., female YOGA-DP instructors for female participants). In fact, as mentioned in the results section, the structure of our intervention evolved during the development process to enhance its uptake and adherence. Similar to many other Yoga interventions, our intervention includes Shithilikarana Vyayama, Asana (standing, sitting, and lying poses), Pranayama, and Dhyana and relaxation practices (43, 44, 52). Surya Namaskar is something additional in our intervention which is not always found in Yoga interventions. It can help in the prevention of T2DM, as it is considered a moderate-intensity activity and burns about 3.8–6.7 kcal/min (16, 17). Second, based on the suggestion of Yoga experts, AUM chanting (under Dhyana and relaxation practices) was not retained on religious grounds. Thus, the acceptance of the intervention could be high among people irrespective of their religious beliefs.

Yoga is a complex intervention, and the MRC guidance on developing and evaluating complex interventions provided the overall framework to develop the intervention (9). In fact, intervention development is the first step, and there are other steps as well, such as feasibility/piloting, evaluation, and implementation. There are questions throughout this guidance which helped us to design the study and to follow a systematic iterative process. It is not a “one size fits all” guidance but a generic one, and thus, not all the questions were relevant in our context. A major challenge was to systematically integrate traditional and western medical systems for T2DM prevention. Any traditional advice which is based on anecdotal (or contradictory) evidence was not included in the intervention. A similar systematic approach could be used to develop other local and cross-cultural health interventions.

The study has several strengths and weaknesses. This is one of the few studies to report the systematic development of a Yoga intervention. Another example is a Yoga-based cardiac rehabilitation (Yoga-CaRe) program which has been developed for secondary prevention of myocardial infarction in India (54). The interventions, study participants, and outcomes are different in the two studies. We followed a systematic process and reviewed the scientific literature as part of the process. In other words, we summarized the heterogeneous contents of successful and relevant Yoga interventions. The process also helped us to reach consensus on this complex intervention. A range of stakeholders (including healthcare, medical, and Yoga experts and practitioners and the public) were involved to explore issues like safety and acceptability of the intervention. The systematic review included only those studies that showed evidence of effectiveness in one of the prespecified outcomes. It should be noted that this was not a typical effectiveness systematic review, and the ultimate aim was to develop an intervention based on previous successful interventions. We also excluded studies if they did not report the Sanskrit name of the Yogic practice. Without the Sanskrit name, the English translation could mean more than one Yogic practice (and sometimes even modified or patented Yoga), and it is difficult to replicate such interventions for multiple reasons. There is a chance of missing a relevant Yogic practice. However, Yoga experts were involved in the next step, and they confirmed the inclusion of all the essential Yogic practices. Second, the Jadad score was calculated as part of the methodological quality assessment of the included RCTs. Apart from its many advantages, as mentioned before, it has disadvantages as well. For example, it is over-simplistic and does not take into account many essential methodological issues, such as the concealment of treatment allocation (55). Therefore, in addition to the Jadad score calculation, we assessed the allocation concealment. Third, due to the accessibility issues in India, we had to compress the video, which affected its quality. However, we have archived the original high-quality video for future use.

A multi-center feasibility RCT is currently in progress in India to determine the feasibility of undertaking the main RCT (56). If the feasibility is acceptable, we will design and conduct the main RCT. If the intervention is found to be effective, it will be a low-cost, acceptable, and local solution for preventing T2DM among high-risk people in India and for improving their overall health. It will also prevent the future clinical, personal, and economic burden of T2DM on patients, their families, the health system, and the economy. The advantages of preventing T2DM may extend to the prevention of T2DM related complications. More evidence-based choices will be available to people for preventing T2DM. The intervention will simultaneously empower people to manage their health. Given that T2DM and its related costs are global concerns and Yoga is popular or is becoming popular globally, there will be worldwide interest in this low-cost Yoga-based T2DM prevention program, particularly in other South Asian countries and in countries with South Asian ethnic minorities (57, 58). The intervention could be adapted, evaluated, and implemented to prevent T2DM in other settings or populations.

In conclusion, we systematically developed a novel Yoga program for T2DM prevention (YOGA-DP) among high-risk people in India. A multi-center feasibility RCT is in progress in India.

## Data Availability Statement

The raw data supporting the conclusions of this article will be made available by the authors, without undue reservation.

## Ethics Statement

The study was approved by four research ethics committees, namely, Faculty of Medicine and Health Sciences, University of Nottingham (UK); Centre for Chronic Disease Control (CCDC, India); Swami Vivekananda Yoga Anusandhana Samsthana (S-VYASA, India); and Bapu Nature Cure Hospital and Yogashram (BNCHY, India). The patients/participants provided their written informed consent to participate in this study.

## Author Contributions

KC conceptualized, designed, and conducted the study with the help of other authors, wrote the first draft of the manuscript, and other authors contributed significantly to the revision of the manuscript. All authors read and approved the final manuscript.

## Conflict of Interest

The authors declare that the research was conducted in the absence of any commercial or financial relationships that could be construed as a potential conflict of interest.
