# Supplementary material for: Development of a Yoga Program for Type-2 Diabetes Prevention (YOGA-DP) Among High-Risk People in India
Source: Front Public Health. 2020 Nov 17;8:548674. doi: 10.3389/fpubh.2020.548674 (PMC7706999; doi:10.3389/fpubh.2020.548674)
Supplement: Supplementary file 1 [file Data_Sheet_1.docx]

Figure S1: Flowchart depicting the search and screening process of systematic reviews (SRs) and randomized controlled trials (RCTs)

(a) SR

Additional records identified through other sources (IndMED, Google Scholar, reference list)
(n=7)

Records identified through PubMed searching
(n=494)

Records excluded
(n=468)

Records screened
(n=501)

Full-text SRs excluded
(n=21)

Not a peer-reviewed SR (1)

Not a SR (3)

Not a SR on effectiveness (3)

No eligible RCT found (14)

Full-text potential SRs assessed for eligibility
(n=33)

Full-text RCTs excluded
(n=58)

Related to study design e.g., not an/unclear RCT (21)

Related to population e.g., different target population (2)

Related to intervention e.g., not the main/only intervention, no/inadequate description of intervention, modified/patented Yoga (17)

Related to changes in blood glucose levels e.g., not an outcome, no information, no statistically significant improvement (14)

Combination of the above four (3)

Full-text not available even after contacting the corresponding author (1)

SRs included containing potential RCTs
(n=12)

Full-text potential RCTs from SRs assessed for eligibility

(n=67)

RCTs from SRs included

(n=8)

(2 similar articles from the same RCT published in 2 different journals)

(b) RCT

Records identified through PubMed searching
(n=384)

Additional records identified through other sources (IndMED, Google Scholar, reference list)
(n=0)

Records screened
(n=384)

Records excluded
(n=374)

Full-text RCTs excluded
(n=9)

Related to study design e.g., not an RCT + related to population e.g., different target population (1)

Related to intervention e.g., not the main/only intervention, inadequate description of intervention (2)

Related to changes in blood glucose levels e.g., not an outcome, no statistically significant improvement (6)

Full-text potential RCTs assessed for eligibility
(n=10)

RCT included

(n=1)

Table S1: Excluded studies (systematic reviews (SRs) and randomized controlled trials (RCTs)) with reasons for exclusion

| **(a) SR**  ***Full-text SRs excluded (n=21)***  **Not a peer-reviewed SR (1)**  Cosman J. Yoga and diabetes: a systematic review of recent literature concerning the impact of yoga practice on diabetic symptoms. USA: University of Texas Health Science Center. 2014. <https://digitalcommons.library.tmc.edu/dissertations/AAI1599374> [dissertation].  **Not a SR (3)**  Pandey A, Tripathi P, Pandey R, et al. Alternative therapies useful in the management of diabetes: a systematic review. J Pharm Bioallied Sci. 2011;3(4):504-12.  Apor P. Preventive and curative value of yoga in cardiometabolic diseases. Orv Hetil. 2016;157(9):323-7.  Prajapati S, Vyas G, Vyas G. Yoga practice for the prevention and management of diabetes mellitus: a systematic review. Research and Reviews: Journal of Microbiology and Virology. 2017;7(1):31-5.  **Not a SR on effectiveness (3)**  Cramer H, Lauche R, Dobos G. Characteristics of randomized controlled trials of yoga: a bibliometric analysis. BMC Complement Altern Med. 2014;14:328.  McCall MC. In search of yoga: research trends in a western medical database. Int J Yoga. 2014;7(1):4-8.  Jeter PE, Slutsky J, Singh N, et al. Yoga as a therapeutic intervention: a bibliometric analysis of published research studies from 1967 to 2013. J Altern Complement Med. 2015;21(10):586-92.  **No eligible RCT found (14)**  Jayasinghe SR. Yoga in cardiac health (a review). Eur J Cardiovasc Prev Rehabil. 2004;11(5):369-75.  Innes KE, Bourguignon C, Taylor AG. Risk indices associated with the insulin resistance syndrome, cardiovascular disease, and possible protection with yoga: a systematic review. J Am Board Fam Pract. 2005;18(6):491-519.  Innes KE, Vincent HK. The influence of yoga-based programs on risk profiles in adults with type 2 diabetes mellitus: a systematic review. Evid Based Complement Alternat Med. 2007;4(4):469-86.  Pilkington K, Stenhouse E, Kirkwood G, et al. Diabetes and complementary therapies: mapping the evidence. Practical Diabetes International. 2007;24(7):371-6.  Yang K. A review of yoga programs for four leading risk factors of chronic diseases. Evid Based Complement Alternat Med. 2007;4(4):487-91.  Anderson JG, Taylor AG. The metabolic syndrome and mind-body therapies: a systematic review. J Nutr Metab. 2011;2011:276419.  Sharma M, Knowlden AP. Role of yoga in preventing and controlling type 2 diabetes mellitus. J Evid Based Complementary Altern Med. 2012;17(2):88-95.  Rioux JG, Ritenbaugh C. Narrative review of yoga intervention clinical trials including weight-related outcomes. Altern Ther Health Med. 2013;19(3):32-46.  Thent ZC, Das S, Henry LJ. Role of exercise in the management of diabetes mellitus: the global scenario. PLoS One. 2013;8(11):e80436.  Hartley L, Dyakova M, Holmes J, et al. Yoga for the primary prevention of cardiovascular disease. Cochrane Database Syst Rev. 2014;(5):CD010072.  Barrows JL, Fleury J. Systematic review of yoga interventions to promote cardiovascular health in older adults. West J Nurs Res. 2016;38(6):753-81.  Cramer H, Langhorst J, Dobos G, et al. Yoga for metabolic syndrome: a systematic review and meta-analysis. Eur J Prev Cardiol. 2016;23(18):1982-93.  Cai H, Li G, Zhang P, et al. Effect of exercise on the quality of life in type 2 diabetes mellitus: a systematic review. Qual Life Res. 2017;26(3):515-30.  Haider T, Sharma M, Branscum P. Yoga as an alternative and complimentary therapy for cardiovascular disease: a systematic review. J Evid Based Complementary Altern Med. 2017;22(2):310-6.  ***Full-text RCTs excluded (n=58)***  **Related to study design e.g., not an/unclear RCT (21)**  Jain SC, Uppal A, Bhatnagar SO, et al. A study of response pattern of non-insulin dependent diabetics to yoga therapy. Diabetes Res Clin Pract. 1993;19(1):69-74.  Jain SC, Talukdar B. Role of yoga in control of hyperglycemia in middle aged patients of non-insulin dependent diabetes mellitus. Indian J Clin Biochem. 1995;10(2):62-5.  Malhotra V, Singh S, Singh KP, et al. Study of yoga asanas in assessment of pulmonary function in NIDDM patients. Indian J Physiol Pharmacol. 2002;46(3):313-20.  Malhotra V, Singh S, Tandon OP, et al. Effect of yoga asanas on nerve conduction in type 2 diabetes. Indian J Physiol Pharmacol. 2002;46(3):298-306.  Singh S, Malhotra V, Singh KP, et al. Role of yoga in modifying certain cardiovascular functions in type 2 diabetic patients. J Assoc Physicians India. 2004;52:203-6.  Malhotra V, Singh S, Tandon OP, et al. The beneficial effect of yoga in diabetes. Nepal Med Coll J. 2005;7(2):145-7.  Bijlani RL, Vempati RP, Yadav RK, et al. A brief but comprehensive lifestyle education program based on yoga reduces risk factors for cardiovascular disease and diabetes mellitus. J Altern Complement Med. 2005;11(2):267-74.  Gordon L, Morrison EY, McGrowder DA, et al. Changes in clinical and metabolic parameters after exercise therapy in patients with type 2 diabetes. Arch Med Sci. 2008;4(4):427-37.  Gordon LA, Morrison EY, McGrowder DA, et al. Effect of exercise therapy on lipid profile and oxidative stress indicators in patients with type 2 diabetes. BMC Complement Altern. 2008;8:21-30.  Mahapure HH, Shete SU, Bera TK. Effect of yogic exercise on super oxide dismutase levels in diabetics. Int J Yoga. 2008;1(1):21-6.  Singh S, Kyizom T, Singh KP, et al. Influence of pranayamas and yoga-asanas on serum insulin, blood glucose and lipid profile in type 2 diabetes. Indian J Clin Biochem. 2008;23(4):365-8.  Malhotra V, Singh S, Sharma SB, et al. The status of NIDDM patients after yoga asanas: assessment of important parameters. J Clin Diagn Res. 2010;4(3):2652-67.  Kyizom T, Singh S, Singh KP, et al. Effect of pranayama and yoga-asana on cognitive brain functions in type 2 diabetes: P3 event related evoked potential (ERP). Indian J Med Res. 2010;131(5):636-40.  Balaji PA, Smitha RV, Syed SA. Effects of yoga-pranayama practices on metabolic parameters and anthropometry in type 2 diabetes. Int Multidiscip Res J. 2011;1(10):1-4.  Hegde SV, Adhikari P, Kotian S, et al. Effect of 3-month yoga on oxidative stress in type 2 diabetes with or without complications: a controlled clinical trial. Diabetes care. 2011;34(10):2208-10.  Madanmohan, Bhavanani AB, Dayanidy G, et al. Effect of yoga therapy on reaction time, biochemical parameters and wellness score of peri and post-menopausal diabetic patients. Int J Yoga. 2012;5(1):10-5.  Beena RK, Sreekumaran E. Yogic practice and diabetes mellitus in geriatric patients. Int J Yoga. 2013;6(1):47-54.  Bindra M, Nair S, Darotiya S. Influence of pranayamas and yoga-asanas on blood glucose, lipid profile and HbA1c in type 2 diabetes. Int J Pharma Bio Sci. 2013;4(1):169-72.  Dash S, Thakur AK. Effect of yoga in patient's with type-II diabetes mellitus. J Evol Med Dent Sci. 2014;3(7):1642-56.  Popli U, Subbe CP, Sunil K. The role of yoga as a lifestyle modification in treatment of diabetes mellitus: results of a pilot study. Altern Ther Health Med. 2014;20(6):24-6.  Yadav R, Yadav RK, Pandey RM, et al. Effect of a short-term yoga-based lifestyle intervention on health-related quality of life in overweight and obese subjects. J Altern Complement Med. 2016;22(6):443-9.  **Related to population e.g., different target population (2)**  Lee JA, Kim JW, Kim DY. Effects of yoga exercise on serum adiponectin and metabolic syndrome factors in obese postmenopausal women. Menopause. 2012;19(3):296-301.  Seo DY, Lee S, Figueroa A, et al. Yoga training improves metabolic parameters in obese boys. Korean J Physiol Pharmacol. 2012;16(3):175-80.  **Related to intervention e.g., not the main/only intervention, no/inadequate description of intervention, modified/patented Yoga (17)**  Monroe R, Power J, Kumar A, et al. Yoga therapy for NIDDM: a controlled trial. Complementary Medicine Journal. 1992;6:66-8.  Céspedes EM, Riverón G, Alonso CA, et al. Evolución metabólica de pacientes diabéticos tipo 2 sometidos a un tratamiento combinado de dieta y ejercicios yoga. Revista Cubana de Investigaciones Biomedicas. 2002;21(2):98-101.  Elder C, Aickin M, Bauer V, et al. Randomized trial of a whole-system ayurvedic protocol for type 2 diabetes. Altern Ther Health Med. 2006;12(5):24-30.  Khatri D, Mathur KC, Gahlot S, et al. Effects of yoga and meditation on clinical and biochemical parameters of metabolic syndrome. Diabetes Res Clin Pract. 2007;78(3):e9-10.  Gordon L, Morrison EY, McGrowder D, et al. Effect of yoga and traditional physical exercise on hormones and percentage insulin binding receptor in patients with type 2 diabetes. Am J Biochem Biotechnol. 2008;4(1):35-42.  Pardasany A, Shenoy S, Sandhu JS. Comparing the efficacy of tai chi chuan and hatha yoga in type 2 diabetes mellitus patients on parameters of blood glucose control and lipid metabolism. Indian J Physiother Occup Ther. 2010;4(3):11-6.  Singh H, Sharma A, Johar S. Comparative clinical evaluation of the antidyslipidaemic effects of lashunadi compound and yogic exercises in patients of metabolic syndrome. Indian Journal of Traditional Knowledge. 2011;10(4):651-6.  Jyotsna VP, Joshi A, Ambekar S, et al. Comprehensive yogic breathing program improves quality of life in patients with diabetes. Indian J Endocrinol Metab. 2012;16(3):423-8.  Čokolič M, Herodež ŠS, Sternad S, et al. The inhibitory effect of laughter yoga on the increase in postprandial blood glucose in type 2 diabetic patients. Diabetol Croat. 2013;42(2):54-8.  Habibi N, Farsani ZH, Yazdani B, et al. The influence of yoga-on risk profiles programs in women with diabetes type II. Adv Environ Biol. 2013;7(4):550-6.  Jyotsna VP, Ambekar S, Singla R, et al. Cardiac autonomic function in patients with diabetes improves with practice of comprehensive yogic breathing program. Indian J Endocrinol Metab. 2013;17(3):480-5.  Rast SD, Hojjati Z, Shabani R. The effect of yoga training on lipid profile and blood glucose in type II diabetic females. Ann Biol Res. 2013;4(8):128-33.  Habibi N, Marandi SM. Effect of 12 weeks of yoga practice on glucose, insulin and triglycerides serum level in women with diabetes type II. J Gorgan Univ Med Sci. 2014;15(4):1-7.  Jyotsna VP, Dhawan A, Sreenivas V, et al. Completion report: effect of comprehensive yogic breathing program on type 2 diabetes: a randomized control trial. Indian J Endocrinol Metab. 2014;18(4):582-4.  Kanaya AM, Araneta MR, Pawlowsky SB, et al. Restorative yoga and metabolic risk factors: the practicing restorative yoga vs. stretching for the metabolic syndrome (PRYSMS) randomized trial. J Diabetes Complications. 2014;28(3):406-12.  Rast S, Hojjati Z, Shabani R. The effect of yoga training on blood glucose, insulin and resting heart rate in type II diabetic females. Res J Sport Sci. 2014;2(1):15-21.  Balaji PV, Thirumaran M. Effects of 10 weeks yoga training on blood glucose and lipid profile in type II diabetic patients. Sch J App Med Sci. 2015;3(5):1876-9.  **Related to changes in blood glucose levels e.g., not an outcome, no information, no statistically significant improvement (14)**  Cohen BE, Chang AA, Grady D, et al. Restorative yoga in adults with metabolic syndrome: a randomized, controlled pilot trial. Metab Syndr Relat Disord. 2008;6(3):223-9.  Amita S, Prabhakar S, Manoj I, et al. Effect of yoga-nidra on blood glucose level in diabetic patients. Indian J Physiol Pharmacol. 2009;53(1):97-101.  Yang K, Bernardo LM, Sereika SM, et al. Utilization of 3-month yoga program for adults at high risk for type 2 diabetes: a pilot study. Evid Based Complement Alternat Med. 2011;2011:257891.  Shantakumari N, Sequeira S, Eldeeb R. Effect of a yoga intervention on hypertensive diabetic patients. Journal of Advances in Internal Medicine. 2012;1(2):60-3.  Subramaniyan TG, Subramaniyan N, Chidambaram M. Brisk walking and yoga as adjuvant therapy in management of type 2 diabetes mellitus. International Journal of Students' Research. 2012;2(1):43-6.  Hegde SV, Adhikari P, Shetty S, et al. Effect of community-based yoga intervention on oxidative stress and glycemic parameters in prediabetes: a randomized controlled trial. Complement Ther Med. 2013;21(6):571-6.  Kim HN, Ryu J, Kim KS, et al. Effects of yoga on sexual function in women with metabolic syndrome: a randomized controlled trial. J Sex Med. 2013;10(11):2741-51.  Manchanda SC, Mehrotra UC, Makhija A, et al. Reversal of early atherosclerosis in metabolic syndrome by yoga: a randomized controlled trial. J Yoga Phys Ther. 2013;3(1):1-3.  Shantakumari N, Sequeira S. Effects of a yoga intervention on lipid profiles of diabetes patients with dyslipidemia. Indian Heart J. 2013;65(2):127-31.  Corey SM, Epel E, Schembri M, et al. Effect of restorative yoga vs. stretching on diurnal cortisol dynamics and psychosocial outcomes in individuals with the metabolic syndrome: the PRYSMS randomized controlled trial. Psychoneuroendocrinology. 2014;49:260-71.  Kumar MU, Kalidasan R. Influences of yogic practises on blood glucose and lipid profile among male type 2 diabetes patients and lipid profile among male type 2 diabetes patients. International Journal of Multidisciplinary Educational Research. 2014;3(4(3)):202-13.  McDermott KA, Rao MR, Nagarathna R, et al. A yoga intervention for type 2 diabetes risk reduction: a pilot randomized controlled trial. BMC Complement Altern Med. 2014;14(1):212-25.  Giri M, Artanayasa W, Putra A. Effect of yoga on atherosclerosis risk in type 2 diabetes. **Proceeding ICIRAD**. 2015;1(1):141.  Siu PM, Angus PY, Benzie IF, et al. Effects of 1-year yoga on cardiovascular risk factors in middle-aged and older adults with metabolic syndrome: a randomized trial. Diabetol Metab Syndr. 2015;7(1):40-51.  **Combination of the above four (3)**  Kerr D, Gillam E, Ryder J, et al. An eastern art form for a western disease: randomised controlled trial of yoga in patients with poorly controlled insulin-treated diabetes. Practical Diabetes International. 2002;19(6):164-6.  Agte VV, Tarwadi K. Sudarshan kriya yoga for treating type 2 diabetes: a preliminary study. Altern Complement Ther. 2004;10(4):220-2.  Skoro-Kondza L, Tai SS, Gadelrab R, et al. Community based yoga classes for type 2 diabetes: an exploratory randomised controlled trial. BMC Health Serv Res. 2009;9(1):1-8.  **Full-text not available even after contacting the corresponding author (1)**  Anitha S, Moses CRA, Judie A. Yoga therapy on quality of life, selected physiological and bio-chemical parameters of patients with type 2 diabetes mellitus. The Journal of Nursing Trendz. 2013;4(2):3-8.  **(b) RCT**  ***Full-text RCTs excluded (n=9)***  **Related to study design e.g., not an RCT + related to population e.g., different target population (1)**  Wolff M, Memon AA, Chalmers JP, et al. Yoga’s effect on inflammatory biomarkers and metabolic risk factors in a high risk population: a controlled trial in primary care. BMC Cardiovasc Disord. 2015;15:91.  **Related to intervention e.g., not the main/only intervention, inadequate description of intervention (2)**  Mullur RS, Ames D. Impact of a 10 minute seated yoga practice in the management of diabetes. J Yoga Phys Ther. 2016;6(1):1000224.  Nagasukeerthi P, Mooventhan A, Manjunath NK. Short-term effect of add on bell pepper (Capsicum annuum var. grossum) juice with integrated approach of yoga therapy on blood glucose levels and cardiovascular functions in patients with type 2 diabetes mellitus: a randomized controlled study. Complement Ther Med. 2017;34:42-5. {Nagasukeerthi P, Mooventhan A, Manjunath NK. Corrigendum to "Short-term effect of add on bell pepper (Capsicum annuum var. grossum) juice with integrated approach of yoga therapy on blood glucose levels and cardiovascular functions in patients with type 2 diabetes mellitus: a randomized controlled study.” [Comp. Ther. Med. 34 (2017) 42-45]. Complement Ther Med. 2018;37:185.}    **Related to changes in blood glucose levels e.g., not an outcome, no statistically significant improvement (6)**  Sohl SJ, Wallston KA, Watkins K, et al. Yoga for risk reduction of metabolic syndrome: patient-reported outcomes from a randomized controlled pilot study. Evid Based Complement Alternat Med. 2016;2016:3094589.  Ebrahimi M, Guilan-Nejad TN, Pordanjani AF. Effect of yoga and aerobics exercise on sleep quality in women with type 2 diabetes: a randomized controlled trial. Sleep Sci. 2017;10(2):68-72.  Sreedevi A, Unnikrishnan AG, Karimassery SR, et al. A randomized controlled trial of the effect of yoga and peer support on glycaemic outcomes in women with type 2 diabetes mellitus: a feasibility study. BMC Complement Altern Med. 2017;17(1):100.  Sreedevi A, Unnikrishnan AG, Karimassery SR, et al. The effect of yoga and peer support interventions on the quality of life of women with diabetes: results of a randomized controlled trial. Indian J Endocrinol Metab. 2017;21(4):524-30.  Schmid AA, Atler KE, Malcolm MP, et al. Yoga improves quality of life and fall risk-factors in a sample of people with chronic pain and type 2 diabetes. Complement Ther Clin Pract. 2018;31:369-73.  Supriya R, Yu AP, Lee PH, et al. Yoga training modulates adipokines in adults with high-normal blood pressure and metabolic syndrome. Scand J Med Sci Sports. 2018;28(3):1130-8. |
| --- |

Table S2: Study characteristics of the included nine RCTs

| Author and year | In systematic review and year | Location | Population | Sample size | Intervention | Comparator | Improvement in blood glucose levels in the Yoga group (from baseline to follow-up^) | Adverse events |
| --- | --- | --- | --- | --- | --- | --- | --- | --- |
| Agrawal 2003 | Aljasir 2010, Cramer 2014, Innes 2016, Vizcaino 2016, Eun 2017, Kumar 2016, Thind 2017 | Bikaner, India | T2DM, both sexes | 200 (I=100?, C=100?) | Standard medical management?+Yoga | Standard medical management+exercise? | FBG (from 183.9±39.3 to 150.7±34.2 mg/dl), HbA1c (from 9.4±1.0 to 8.6±1.0 %) | Yes* |
| Nagarathna 2012 | Cramer 2014, Innes 2016, Vizcaino 2016, Cui 2017, Kumar 2016, Thind 2017, Jayawardena 2018 | Bangalore, India | T2DM, both sexes, above 25 years? | 277 (I=141, C=136) | Standard medical management?+Yoga | Standard medical management?+exercise and walking | HbA1c (from 8.5±1.7 to 7.3±3.0 %) | Yes* |
| Vaishali 2011, 2012 | Cramer 2014, Bhurji 2016, Chu 2016, Innes 2016, Pai 2016, Vizcaino 2016, Cui 2017, Kumar 2016, Thind 2017, Jayawardena 2018 | Mangalore, India | T2DM with cardiometabolic risk factors, both sexes, above 60 years | 60 (I=30, C=30) | Standard medical management+Yoga | Standard medical management | FBG (from 163.5±14.8 to 115.6±13.7 mg/dl), HbA1c (from 10.3±0.9 to 9.1±0.6 %) | Yes* |
| Yadav 2013 | Kumar 2016 | Gwalior, India | T2DM, male, 30-55 years | 30 (I=15, C=15) | Yoga | NS | FBG (from 234.5±46.9 to127.7±15.2 mg/dl?) | NS |
| Kumar 2014 | Thind 2017, Vizcaino 2016 | Salem, India | T2DM, male, 35-60 years | 30 (I=15, C=15) | Standard medical management+Yoga | Standard medical management | FBG (from 309.5±19.3 to 287.9±19.9 mg/dl) | NS |
| Kumpatla 2015 | Thind 2017 | Chennai, India | T2DM, both sexes, 25-60 years | 303 (I=154, C=149) | Standard medical management+Yoga | Standard medical management | HbA1c (from 10.6±2.5 to 7.2±1.4 %) | None |
| Sharma 2015 | Thind 2017, Vizcaino 2016 | Jaipur, India | T2DM, both sexes, 35-55 years | 80 (I-40, C=40) | Standard medical management+Yoga | Standard medical management | FBG (from 139.2±16.6 to 99.8±15.9  mg/dl), PPBG (from 174.1±7.9 to 141.7±8.4  mg/dl), HbA1c (from 9.3±1.0 to 7.1±0.7 %) | NS |
| Singh 2015 | Vizcaino 2016 | Gangtok?, India | T2DM, both sexes, above 20 years | 337 (I-Yoga=112, I-music therapy=110, C=115) | Standard medical management+Yoga,  Standard medical management+music therapy | Standard medical management | FBG (from 166.9±6.1 to 140.0±3.2 mg/dl), PPBG (from 214.1±7.6 to 173.3±5.9 mg/dl), HbA1c (from 8.6±0.6 to 7.6±0.6 %) | Yes* |
| Keerthi 2017 |  | Puducherry, India | Prediabetes and T2DM with normal BP, both sexes, 18-45 years | 310 (Healthy with normal BP: C=62; Prediabetes with normal BP: I=62, C=62; T2DM with normal BP: I=62, C=62) | Prediabetes and T2DM with normal BP: standard medical management+Yoga | Healthy with normal BP: no change in lifestyle; Prediabetes and T2DM with normal BP: standard medical management+walking | Prediabetes with normal BP- FBG (from 112.2±7.6 to 83.4±12.9 mg/dl);  Diabetes with normal BP- FBG (from 195.4±49.9 to 152.3±31.3 mg/dl) | NS |

NS=Not specified

*Limited information

^Mean±standard deviation

?Unclear

Table S3: Critical appraisal of the included nine RCTs

| Author and year | Randomization method | Allocation concealment | Blinding of outcome assessors | Withdrawals and dropouts | Jadad score |
| --- | --- | --- | --- | --- | --- |
| Agrawal 2003 | NS | NS | NS | Yes | Low |
| Nagarathna 2012 | Yes | Yes | Yes | Yes | High |
| Vaishali 2011, 2012 | Yes* | Yes* | NS | Yes | High |
| Yadav 2013 | NS | NS | NS | NS | Low |
| Kumar 2014 | NS | NS | NS | NS | Low |
| Kumpatla 2015 | Yes± | NS | NS | Yes* | Low± |
| Sharma 2015 | NS | NS | Yes | NS | Low |
| Singh 2015 | Yes | Yes | NS^ | Yes | High |
| Keerthi 2017 | Yes* | Yes* | Yes | Yes* | High |

NS=Not specified

*Limited information

±Not true randomization

^Data analyst was blind
